# Mechanisms of protective immune responses induced by the *Plasmodium falciparum* circumsporozoite protein-based, self-assembling protein nanoparticle vaccine

**DOI:** 10.1186/1475-2875-12-136

**Published:** 2013-04-22

**Authors:** Margaret E McCoy, Hannah E Golden, Tais APF Doll, Yongkun Yang, Stephen A Kaba, Peter Burkhard, David E Lanar

**Affiliations:** 1 Malaria Vaccine Branch, WRAIR, 503 Robert Grant Avenue, Silver Spring, MD 20910, USA; 2 Institute of Materials Science, University of Connecticut, 97 North Eagleville Road, Storrs, CT 06269, USA; 3 Department of Molecular and Cell Biology, University of Connecticut, 97 North Eagleville Road, Storrs, CT 06269, USA

**Keywords:** Self-assembling protein nanoparticle, *Plasmodium*, Vaccine, Erythrocytic stage, Circumsporozoite protein, TAP, Dendritic cells, Complement lysis

## Abstract

**Background:**

A lack of defined correlates of immunity for malaria, combined with the inability to induce long-lived sterile immune responses in a human host, demonstrate a need for improved understanding of potentially protective immune mechanisms for enhanced vaccine efficacy. Protective sterile immunity (>90%) against the *Plasmodium falciparum* circumsporozoite protein (CSP) has been achieved using a transgenically modified *Plasmodium berghei* sporozoite (Tg-*Pb*/*Pf*CSP) and a self-assembling protein nanoparticle (SAPN) vaccine presenting CSP epitopes (*Pf*CSP-SAPN). Here, several possible mechanisms involved in the independently protective humoral and cellular responses induced following SAPN immunization are described.

**Methods:**

Inbred mice were vaccinated with *Pf*CSP-SAPN in PBS. Serum antibodies were harvested and effects on *P*. *falciparum* sporozoites mobility and integrity were examined using phase contrast microscopy. The functionality of SAPN-induced antibodies on inhibition of sporozoite invasion and growth within primary human hepatocytes was also examined. The internal processing of SAPN by bone marrow-derived dendritic cells (BMDDC), using organelle-specific, fluorescent-tagged antibody or gold-encapsulated SAPN, was observed using confocal or electron microscopy, respectively.

**Results:**

The results of this work demonstrate that *Pf*CSP-SAPN induces epitope-specific antibody titers, predominantly of the Th2 isotype IgG1, and that serum antibodies from PfCSP-SAPN-immunized mice appear to target *P*. *falciparum* sporozoites via the classical pathway of complement. This results in sporozoite death as indicated by cessation of motility and the circumsporozoite precipitation reaction. Moreover, *Pf*CSP-SAPN-induced antibodies are able to inhibit wild-type *P*. *falciparum* sporozoite invasion and growth within cultured primary human hepatocytes. In addition, the observation that *Pf*CSP-SAPN are processed (and presented) to the immune system by dendritic cells in a slow and continuous fashion via transporter associated with antigen processing (TAP) recruitment to the early endosome (EE), and have partially delayed processing through the endoplasmic reticulum, has the potential to induce the long-lived, effector memory CD8^+^ T-cells as described previously.

**Conclusion:**

This paper describes the examination of humoral and cellular immune mechanisms induced by *Pf*CSP-SAPN vaccination which result in sterile host protection against a transgenic *P*. *berghei* malaria sporozoite expressing the *P*. *falciparum* CSP, and which significantly inhibits native *P*. *falciparum* sporozoites from invading and developing within cultured human hepatocytes. These results may indicate the type and mode of action of protective antibodies needed to control *P*. *falciparum* sporozoites from infecting humans as well as a potential mechanism of induction of protective long-lived effector memory CD8^+^ T-cells.

## Background

The most basic and desirable outcome of a successful vaccine is that it will induce sterile and long-lived immunity in the host. Historically, with most vaccines, this result has been best achieved through the use of chemically fixed, heat-killed or live-attenuated organisms. In the case of malaria this approach has been relatively unsuccessful but more interest has recently been dedicated to these methods [1-3]. Recombinant subunit protein vaccines targeting various parasite proteins of choice, used in combination with immune-boosting adjuvants, have been examined and have yielded promising but limited results. The vaccine RTS,S, currently in Phase 3 human trials, is a subunit circumsporozoite protein (CSP) linked to the Hepatitis B surface protein (HBSP) which self-assembles with native HBSP to form a virus-like particle. RTS,S vaccination has resulted in somewhat reduced clinical infection [4,5]. Longevity studies examining the observed reduced parasite loads, as well as more detailed investigations into possible correlates of this protection, will hopefully be forthcoming in the next few years to further evaluate this vaccine delivery platform.

Malaria vaccinology suffers from a lack of understanding of viable mechanisms by which the host’s immune system is able to efficiently respond to such a highly polymorphic parasite. Even naturally acquired immunity is slow to develop and is easily lost [6]. There is little evidence to support correlations of vaccine-induced human *Plasmodium* immunity or sterile protection from vaccines for malaria. If we could understand, induce and/or manipulate effective mechanisms that would lead to complete protection and long-lived immunity against *Plasmodium* infection, we may be better armed to improve on or design novel vaccine platforms that could enhance host immunity to this end. The studies presented here are an investigation into the mechanisms behind the efficacy of a novel type of immunogen, a self-assembling protein nanoparticle (SAPN) [7-9]. These SAPN have been successfully used to deliver *Plasmodium falciparum* CSP-derived T- and B-cell epitopes to generate a protective immune response against malaria, which is believed to act, in part, by enhanced repetitive display of highly immunogenic peptides [10,11].

The innate immune system can be a critical player in effective immunity to malaria infection [12]. Innate mechanisms of protection are the first and most non-specific immunity the host has in its arsenal against a primary infection. These initial mechanisms link and relate tailored responses that are required to properly and effectively protect against secondary infections. Other than the use of some poorly understood classes of adjuvants, little is known about the specific initial responses required, in conjunction with vaccine administration, to provide complete protection against human malaria infection. The innate system, targeted in synergy with adaptive immune responses, can often outweigh the immunological importance of either in isolation [12]. Various vaccines have demonstrated potential roles for non-specific mediators such as secreted factors and cells in managing malaria infection [13-16], but more may be required from both branches of the immune system in terms of understanding and promoting “cross-talk” between branches to achieve an efficacious vaccine product against human malaria.

To improve this understanding, and enhance an awareness of potential avenues for boosting vaccine efficacy, this paper examines several interactions between innate and adaptive immunity following SAPN immunization. The results show that SAPN-induced antibodies exhibit an ability to inhibit motility and induce complement (C’) lysis of malaria parasites prior to liver infection. Moreover, tracking fluorescently labeled or gold-tagged SAPN demonstrate a delayed processing and (presentation) of *Pf*CSP-SAPN by dendritic cells that may help explain the induction of the previously reported highly effective and long-lived adaptive cellular responses [11].

## Methods

### Vaccines

SAPN were synthesized and assembled as previously described [11]. Gold-encapsulating SAPN were synthesized [17] by first denaturing *Pf*CSP-SAPN overnight in 9 M urea, 20 mM HEPES, pH 7.5, 50 mM NaCl, 5% glycerol. Next, the protein monomers were concentrated to 1 mg/ml using Amicon MWCO 3000. After concentration the monomers were diluted 20-fold into 20 mM HEPES, pH 7.5, 50 mM NaCl, 5% glycerol containing 10 nm citrate-coated gold nanoparticles (Nanocs, Inc via Thermo Fisher Scientific, Inc, Pittsburgh, PA, USA). The final molar ratio of protein chains per gold nanoparticle was 420:1. Dialysis was performed overnight in 20 mM HEPES, pH 7.5, 50 mM NaCl, 5% glycerol to remove the remaining urea. The gold-encapsulating SAPN were characterized by transmission electron microscopy (TEM) and dynamic light scattering (DLS).

*Pf*CSP-SAPN displayed the (NANP) central repeat peptide of the *Pf*CSP; the *Pf*CSP-KMY-SAPN [11] also displayed the central repeat peptide but, in addition, contained 3 CD8^+^ T-cell epitopes from the *Pf* CSP. *Pv*CSP-SAPN contained the same scaffold as *Pf* CSP but displayed the *Plasmodium vivax* CSP central repeat peptide [11].

### Immunizations

Female C57BL/6 mice five to six weeks of age, sex- and age-matched from the Jackson Laboratory (Bar Harbor, ME, USA) were injected ip or im with 10 μg/mouse *Pf*CSP-SAPN or sterile saline, for a total of three immunizations administered two weeks apart. Blood was drawn for antibody analysis one day prior to each immunization and two weeks post final immunization via tail vein nick. All animal protocols were conducted following review and approval by the WRAIR IACUC.

### ELISA

Serum antibody levels were determined following *Pf*CSP-SAPN immunization by ELISA. Anti-*Pf*CSP antibody isotype profiles were determined using IgG1, IgG2c, IgG3 and IgE isotype-specific goat anti-mouse horseradish peroxidase-tagged secondary antibodies (Southern Biotech, Birmingham, AL, USA). Color change was initiated with ABTS Peroxidase Solution (KPL, Gaithersburg, MD, USA) and analysed by absorbance OD_405_ =1.

### Cell culture

Bone marrow was flushed from the femurs of C57BL/6 mice using a 26G needle syringe and cultured for nine days at a concentration of 1.5 × 10^6^ cells/ml in RPMI complete media in the presence of 200 ng/ml recombinant murine Flt3-Ligand (BioVision, Mountain View, CA, USA) and 20 ng/ml recombinant human GM-CSF or 20 ng/ml recombinant human GM-CSF and 20 ng/ml recombinant human IL-4 (both from PeproTech, Inc, Rocky Hill, NJ, USA) in 24 well plates, for a final purity consistent with previous studies using Flt3-Ligand [18]. Cells were maintained and used on day nine or ten of culture and treated/handled as described for flow cytometry and microscopy.

### Flow cytometry

Surface staining of bone marrow-derived dendritic cells (BMDDCs) was performed and evaluated by flow cytometry with antibodies recognizing CD8α, CD11b, CD11c, CD103 and CD207. All antibodies were obtained through BD Biosciences (San Jose, CA, USA) or e-Biosciences (San Diego, CA, USA).

### Confocal microscopy

BMDDCs were harvested from culture, transferred to eight-well chamber slides, and allowed to adhere overnight in the presence of 5 μl early endosome (EE) marker, (Invitrogen, Eugene, OR, USA), 6.67 ng/ml in house-labeled SAV-TAP, and/or 5 × 10^-6^ mmol Lysotracker (Invitrogen, Eugene, OR, USA) in a final staining volume of 2.5-7.5 × 10^5^ cells/150 μl/well. Cells were also co-cultured with 1.5 μg/ml of in-house TAMRA-5-labeled SAPN or ovalbumin at ten-minute, two-hour and overnight time points. Slides were washed, fixed with methanol or 4% PAF and DAPI was added prior to coverslipping. Analysis was performed using confocal microscopy.

### Electron microscopy

BMDDCs were co-cultured with 20–50 μg/ml either in-house gold-encapsulating SAPN ovalbumin or gold alone overnight and then harvested from culture and spun at 300×g for eight minutes. SAPN and ovalbumin samples are processed differently from this step forward. The SAPN cell pellet was resuspended in refrigerated 1% glutaraldehyde, 4% paraformaldehyde fixative for one hour at room temperature. Samples were post-fixed in 2% osmium tetroxide for forty-five minutes, embedded in Epon® embedding media and mounted on copper grids. Ovalbumin cell pellets were resuspended in refrigerated 0.1% glutaraldehyde, 4% paraformaldehyde fixative for one hour at room temperature, embedded in LR white medium, mounted on 200-mesh grids, and were given a secondary labeling of 1:25 anti-ovalbumin (Abcam, Cambridge, MA, USA) and 1:10 colloidal gold-labeling (Insight Genomics, Falls Church, VA, USA). All samples were post-stained with 2% uranyl acetate for twenty minutes and lead citrate for five minutes at room temperature. Samples were then analysed by transmission electron microscopy on a Jeol 100CX Electron Microscope.

### Complement analysis

*Anopheles stephensi* mosquitoes infected with *P*. *falciparum* sporozoites were sacrificed by submersion in 70% ETOH. Salivary glands were dissected and processed using the Ozaki method for sporozoite isolation. 1 × 10^5^ sporozoites were placed in 80% of either: serum from *Pf*CSP-SAPN immunized animals, *Pf*CSP-SAPN immunized mouse serum containing ethylenediaminetetraacetic acid (EDTA) to chelate Ca^2+^, heat inactivated *Pf*CSP-SAPN serum (heated at 56°C for forty-five minutes) or in serum from saline immunized animals in pre-warmed 96-well, round-bottom plates for twenty minutes at 37°C. 5–10 μl culture was placed on sterile microscope slides, coverslipped and analysed by phase contrast microscopy at 20× or 67× magnification.

### Immunofluorescence assay

Room temperature immunofluorescence assay (IFA) slides were processed by adding 25 μl/well of diluted serum sample 1:20 and incubated for one hour at 37°C and then washed three times by soaking slides in a reservoir of 1× PBS. After washing 25 μl/well of the secondary antibody, FITC-labeled goat anti-mouse IgG (Jackson ImmunoResearch Laboratories, Inc, West Grove, PA, USA), was then added at a 1:40 dilution in 1% Evans Blue solution and the slide was incubated for thirty minutes at 37°C. The slide was then washed again three times in 1× PBS as described previously and allowed to air dry. Vectashield (Vector Laboratories, Inc, Burlingame, CA, USA) was then added to each well and slides were analysed under fluorescent microscope.

### Inhibition of liver stage development assay

2 × 10^5^ cyropreserved primary human hepatocytes (CPHH) in 100 μL culture media [19,20] were plated, in triplicate, to confluency onto collagen-coated, eight-chamber LAB-TEK slides and incubated overnight. The next day *P*. *falciparum* sporozoites were collected by salivary gland dissection; 2.5 ×10^5^ sporozoites were incubated at room temperature for twenty minutes with a 1:50 dilution of the indicated serum or with the positive control, NFS-1, a mouse IgG1 monoclonal antibody to *P*. *falciparum* repeats [21] and then added into the wells containing CPHH and incubated at 37°C for three hours to allow sporozoites to infect CPHH. After the three-hour incubation period, CPHH were washed with fresh culture media to remove non-invaded sporozoites. CPHH were harvested on day 4. Upon harvesting, CPHH were trypsinized for fifteen minutes and washed twice: once with fresh media and then with Hank’s balanced salt solution (HBSS). A low speed spin (1,200 rpm for two minutes) was performed between each wash to remove external adherent sporozoites, which remain in the supernatant, and the resulting cell pellet was collected. A quantitative real-time PCR (qRT-PCR) was used to determine the invasion/development rate. For every assay, a standard curve was generated using known numbers of sporozoites (ranging from 4,860 to 20, 1:3 serial dilutions). The qRT-PCR data of each sample were compared to the standard curve and quantified. The invasion/development rate of each test (I_T_) sample is compared to the invasion/development rate of the negative control (I_C_) to give a percent inhibition.

$$\%\mathrm{Inv}\;\mathrm{or}\;\mathrm{Dev}\;=\;\left(I_{c}-I_{T}\right)/I_{c}\times 100$$

## Results

### *Pf*CSP-SAPN-induced antibodies are predominantly Th2 skewed and bind *Plasmodium falciparum* sporozoites

Recently published findings have found that self-assembling protein nanoparticles containing peptide sequences (NANP)_4_ from the central repeat region of the circumsporozoite protein of *P*. *falciparum* induced antibody that was able to sterilely protect mice against lethal challenge [11]. These studies, which looked at the immune response against human malaria *P. falciparum* CSP, were possible to do in a murine model using a transgenic *Plasmodium berghei* sporozoite that has had the full-length circumsporozoite protein (*csp*) gene replaced with that of *P. falciparum*[22]. Thus, *Pf*CSP-SAPN induced antibodies derived in mice are specific for the CSP protein of the human parasite, *P. falciparum*. As IgG1 is a known stimulator of C’ [23], it was undertaken here to demonstrate if the SAPN-induced antibodies were of a particular isotype and if the classical pathway of C’ was involved in protection.

**Figure 1:**
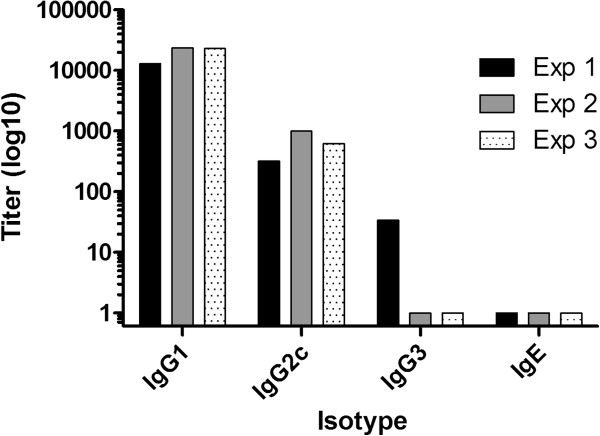
***Pf*CSP-SAPN-induced antibodies are predominantly Th2 skewed.***Pf*CSP-SAPN were given by i.m. injection into C57BL/6 mice and serum was collected two weeks post third immunization and analysed for anti-*Pf*CSP specific antibody isotype titers. Results are shown for three independent experiments, 10 mice per group.

**Figure 2:**
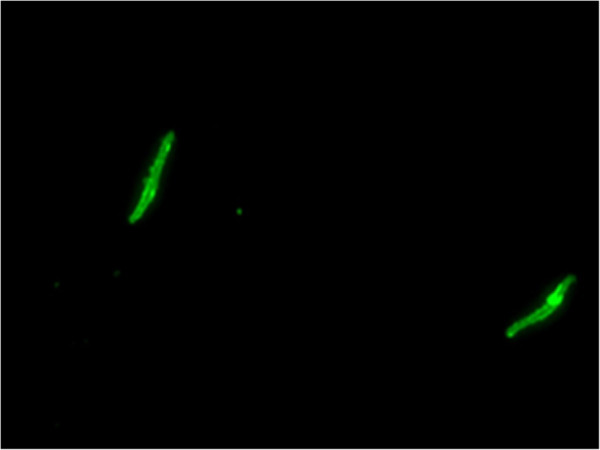
**Immunofluorescence assay of *Plasmodium falciparum *sporozoites incubated with *Pf *CSP-SAPN immune sera at a 1:20 dilution.** Preimmune sera from the mice, sera from *Pv*CSP-SAPN immunized mice, and secondary antibody only sera tests resulted in negative immunofluorescence.

Examination of the antibody isotype profiles derived from SAPN-immunization show a significant predominance of anti-CSP Th2-type IgG1 antibodies (Figure 1). While it was demonstrated that the induction of antibodies that bind to the *Pf*CSP repeat sequence expressed on the genetically modified *P. berghei* parasite, and to a synthetic amino acid sequence bound to a microtiter plate, it was important to determine if the antibodies induced by the SAPN vaccine also bound to CSP on native *P. falciparum* sporozoites This was demonstrated by an IFA (Figure 2). Thus, observing that SAPN-induced antibodies were able to bind *P. falciparum* sporozoites, and with the understanding that IgG1 is able to induce the C’ cascade [23], the question was whether SAPN-induced antibodies would be able to activate C’ and have an effect on *P. falciparum* sporozoites.

### *Pf*CSP-SAPN-induced antibodies are able to alter the morphology of *Plasmodium falciparum* sporozoites and prevent invasion into human hepatocytes

**Figure 3:**
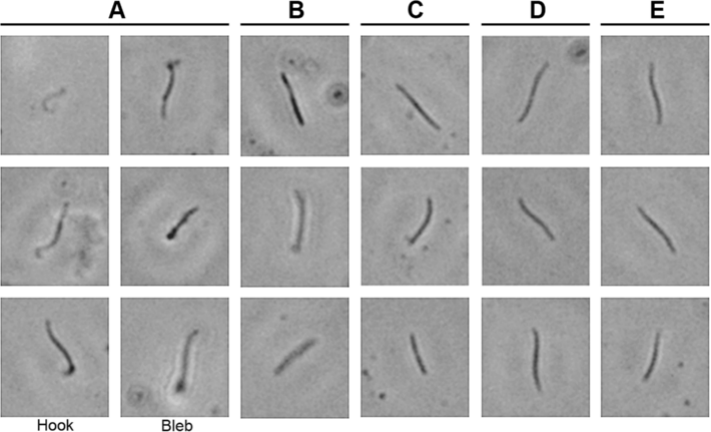
***Plasmodium falciparum *sporozoite morphology is altered by anti-*Pf*CSP-SAPN serum antibodies. ***Plasmodium falciparum* sporozoites were incubated with either *Pf*CSP-SAPN immunized serum taken two weeks post third immunization (Panel **A**) or serum from (Panel **A**) treated to remove Ca^2+^ ions (Panel **B**), serum from (Panel **A**) heat-inactivated in order to inactivate C’ (Panel **C**), serum from PBS-immunized mice (Panel **D**), or serum from mice immunized with *Pv*CSP-SAPN two weeks post third immunization (Panel **E**). Representative images shown; five independent experiments

In order to examine the potential role of antibody-induced C’ induction in sporozoite killing, the morphology of *P. falciparum* sporozoites was examined following co-incubation with or without serum containing CSP-specific antibodies derived from PfCSP-SAPN immunization. Within five minutes of co-incubation it was observed that the immune serum hampered or halted sporozoite motility and phase contrast microscopy demonstrated that after five to twenty minutes of co-incubation there were obvious morphological changes between parasites treated with serum from *Pf*CSP-SAPN immunized mice and control sera (Figure 3, Panels A, D). These changes included the appearance of “blebbing”, or bulbous bulges at the apex of the parasites, “hooked” end phenotypes and elongation and thinning of the parasites. Moreover, if EDTA was added to serum to chelate calcium ions, which are required for the induction of the classical but not the alternative pathway of complement, the normal parasite phenotype was maintained (Figure 3, Panel B). This indicated, specifically, that the classical pathway of complement was involved in the altered morphology and apparent destruction of parasites by *Pf*CSP-SAPN-induced anti-CSP antibodies. Although not sufficient to prove this pathway is solely or uniquely involved in this process, it is a strong indication as to its importance under these conditions. To confirm that without C’ the observed effects on morphology were absent, heat-inactivation of the same serum from *Pf*CSP-SAPN immunized animals was co-incubated with *P. falciparum* sporozoites. After this treatment no alteration of sporozoite phenotype was observed (Figure 3, Panel C). The classical pathway of C’ present in sera, moreover, was not able to affect these changes in morphology in the absence of *Pf*CSP-SAPN-induced antibodies (Figure 3, Panel D), nor did serum from mice immunized with a *Pv*CSP-SAPN [11] presenting the *P. vivax* CSP repeat peptide, (Figure 3, Panel E). These findings, collectively, indicate that *Pf*CSP-SAPN antibodies are specifically able to activate and induce changes in the morphology of *P. falciparum* sporozoites by the induction of the classical pathway of C’.

**Figure 4:**
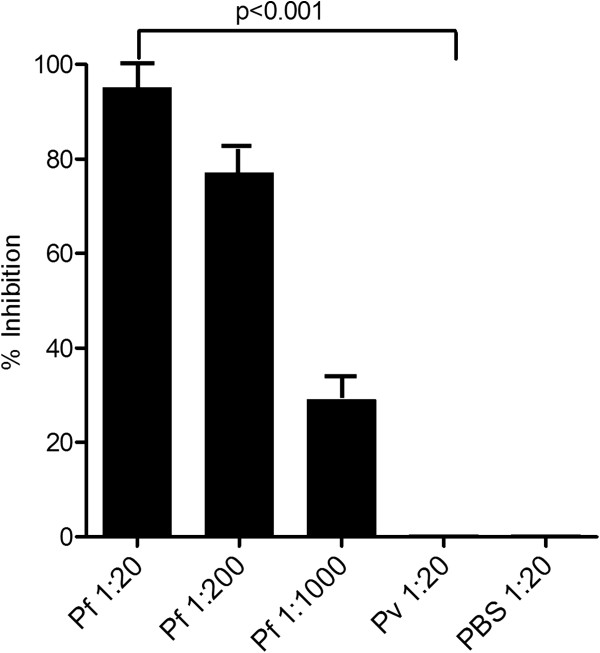
***Pf*CSP-SAPN-induced antibody is able to deter sporozoite liver stage development in a concentration dependent manner.** Human hepatocytes cultured in the presence of *P. falciparum* sporozoites and serum from either *Pf*CSP-SAPN-immunized mice (at 1:20, 1:200, or 1:1,000 dilution), *Pv*CSP-SAPN immunized mice (1:20 dilution) or PBS-sham immunized mice (1:20 dilution). Shown are the reductions in the percent of infected hepatocytes following four days in culture compared to the PBS (1:20 dilution) incubated controls. Error bars are the SD of the mean; *Pf*CSP-SAPN and *Pv*CSP-SAPN at 1:20 dilution show significance using Student’s *t*-test.

To further examine how SAPN-induced CSP-specific antibodies were able to inhibit *P. falciparum* parasites a series of inhibition of liver stage development assays (ILSDA) were performed using primary human hepatocytes. As shown in Figure 4*,* there was an antibody-dependent inhibition of the parasites developing inside the liver cells when sporozoites were exposed to serum from *Pf*CSP-SAPN-immunized mice. This inhibition was not observed when using serum from *Pv*CSP-SAPN or PBS-sham immunized mice. From this test alone it cannot be determined if the resulting reduction of parasites developing inside the liver cells was due to an inhibition of invasion or an impairment of growth after invasion. However, combined with the microscopic observations reported above (Figure 3) of sporozoite morphological changes in the presence of *Pf*CSP-SAPN immune sera, this strongly suggests the reduction in parasite 18S RNA in the liver cells was the result of fewer sporozoites invading the cells after exposure to immune sera.

### Protective adaptive immunity may be the result of delayed antigen processing and presentation of SAPN to CD8+ T-lymphocytes

**Figure 5:**
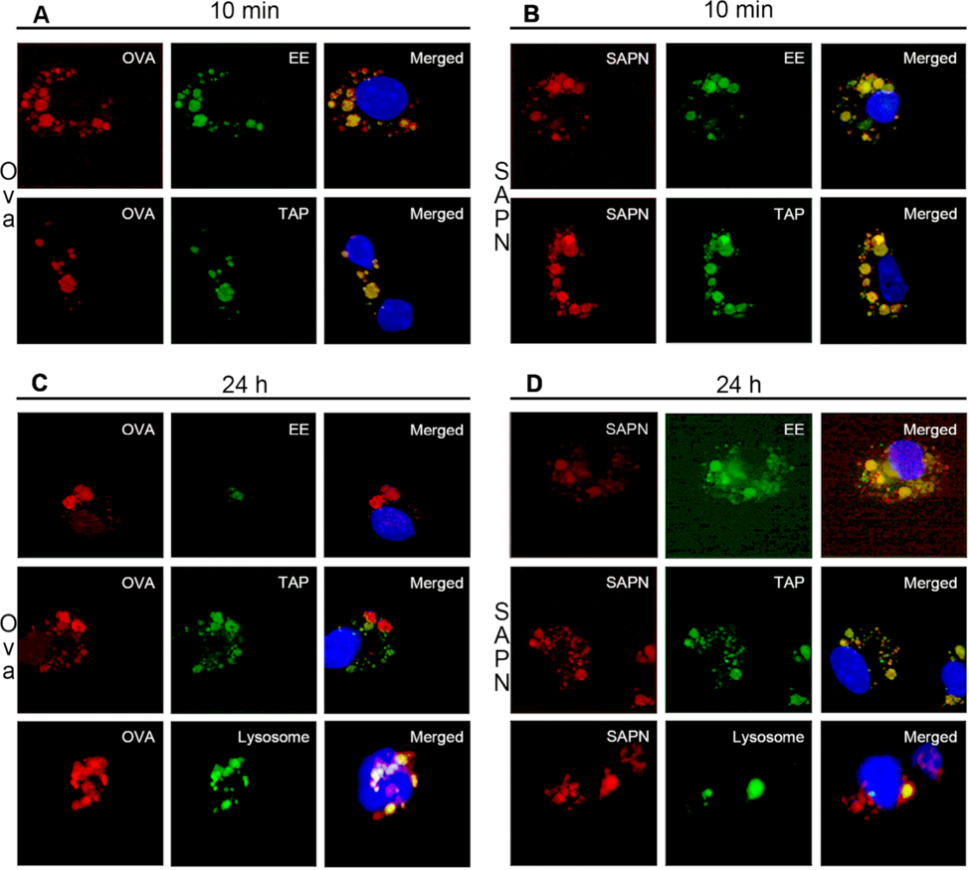
**SAPN have continuous and delayed processing through the early endosome of dendritic cells.** Confocal microscopy images taken at either ten minutes (Panels **A**, **B**) or twenty-four hours (Panels **C**, **D**) following co-culture of bone marrow-derived dendritic cells and either ovalbumin (Panels **A**, **C**) or *Pf*CSP-SAPN (Panels **B**, **D**). Red is labeled Ova or SAPN; green is labeled EE, TAP or lysosomal protein; yellow is the result of merged images of red labeled Ova or SAPN and the green organelle marker dye; blue is the DAPI nuclear stain. Representative images shown; six independent experiments.

**Figure 6:**
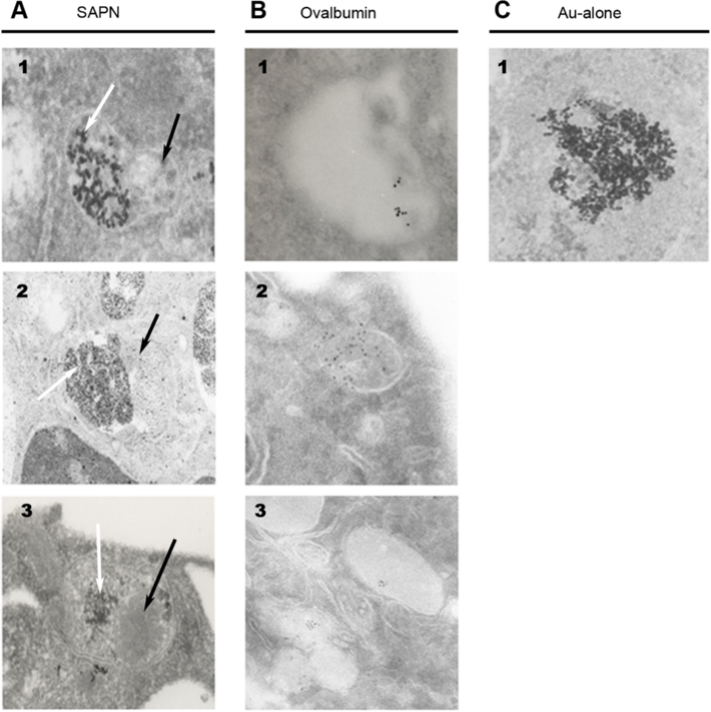
**SAPN have delayed organelle fusion and interaction with lysosomal enzymes.** TEM images after twenty-four hours co-culture of bone marrow-derived dendritic cells and gold-labeled *Pf*CSP-SAPN (Panel **A**), ovalbumin (Panel **B**), or colloidal gold particles alone (Panel **C**). White arrows indicate clusters of gold-encapsulated SAPN; black arrows indicate areas containing a high-density of lysosomal enzymes. 20–67 × magnifications shown. Representative images shown; six independent experiments.

In addition to better understanding the nuances of the humoral responses derived by SAPN immunization, it was also undertaken to understand the way in which *Pf*CSP-SAPN processing and presentation by innate immune cells is able to stimulate the previously observed highly effective and long-lived adaptive cellular responses [11]. By analysing *in vitro* BMDDC by microscopy, the specific kinetics of SAPN processing through BMDDCs could be examined. Results of confocal microscopy show that SAPN have delayed processing in EE organelles, which can last up to twenty-four hours. When compared to a classically processed reference protein, ovalbumin, which is rapidly processed through the endoplasmic reticulum after ten minutes (Figure 5, Panels A-D), the timing is consistent with a cross-presenting mechanism for SAPN, which has the potential to enhance resulting cellular immunity and memory responses. The continuous recruitment of TAP to the EE indicates that SAPN are highly localized (with TAP) for extended time periods at the organelle membrane, which would support the hypothesis of extended loading of SAPN peptides to MHC class I molecules (Figure 5, Panel D). While some SAPN were processed through the lysosome at expected rates, there was a continued processing of SAPN well beyond that of ova in BMDDCs, even up to twenty-four hours (Figure 5, Panels C, D). Differential and delayed processing of SAPN was further confirmed using transmission electron microscopy (TEM), which delineated the segregation of SAPN particles within the late endosome/lysosome fusion organelle up to twenty-four hours following stimulation (Figure 6, Panel A), which was not merely a result of the colloidal gold encapsulation (Figure 6, Panel C). Furthermore, SAPN particles demonstrated delayed interactions with lysosomal enzymes, even though the majority of the reference protein, ova, almost completed its interactions and processing through the organelle at this time point (Figure 6, Panels A, B). Thus this characterization of the ability of SAPN to maintain prolonged processing and loading of antigenic peptides onto MHC class I molecules in the EE via recruitment of TAP, could ultimately offer a substantial enhancement to the induction of long-lived CD8+ T-lymphocyte memory responses [24,25].

## Discussion

The most challenging aspect of designing a vaccine is to understand how to induce efficiently a long-lived and sterilely protective immune response in a host. In the case of malaria, the limited natural host immunity that is observed in endemic areas is short lived if the person leaves the exposure area. Thus, attempts to mimic natural immune responses to infection and develop an effective malaria vaccine over the last quarter century have had significant advances but limited overall success. A major hindrance to this work is a lack of knowledge of the specific correlates of immunity and mechanisms of protection required for malaria infection. Here a novel way of presenting immunogenic protein epitopes to the host’s immune system is described. Evidence for a mechanism of action of a sterilely protective vaccine against malaria using CSP-derived epitopes is presented, and provides insight for an efficacy evaluation of other malaria vaccines.

SAPN-containing peptides derived from the sporozoite CS protein, predicted to stimulate B- and T-lymphocytes, were able to induce robust and long-lived responses against *P. falciparum* in mice [10]. Analysis of the humoral response in this model demonstrated that antibodies produced by SAPN immunization were predominantly skewed towards a Th2 phenotype (IgG1), which has been shown to induce activation of the classical pathway of the complement cascade. The hypothesis that anti-CSP antibodies could bind and alter the morphology of sporozoites was first proposed three decades ago [26], and although there have been several reports which observed the so-called “circumsporozoite protein reaction” or the “circumsporozoite precipitation reaction” [27-29], the specific mechanism behind the observed phenotypic changes was never fully elucidated. The observations of incomplete CSP shedding may be the result of the nature of the antibody isotype and activation of C’ in a very specific fashion. While this study indirectly demonstrates the role of C’ the use of specific inhibitors of C’ activation [30] would be important to further corroborate the importance of the classical pathway in this process of C’ action on sporozoite morphology and function. It was observed here that SAPN-induced antibodies are able to inhibit sporozoite motility, which may either act in conjunction with C’ or by an as yet undetected mechanism independent of C’. This effective concert between innate and adaptive immune responses has the potential to eliminate the majority of sporozoites as they migrate from the site of injection by mosquito to the liver. The potent action of SAPN-immunized serum antibodies, even at low titers [11], indicates that antibodies induced by the SAPN are able to affect shedding of CSP and target the parasite for destruction. This demonstrates that a very high concentration of antibodies may not be a critical requirement for humoral protection of pre-erythrocytic malaria vaccination schemes and suggests that the quality of the antibody is more important than the quantity. In support of this observation, Ferreira *et al.* have shown that 10 μg/ml of antigen-specific antibodies were able to neutralize greater than 98% of injected parasites in a malaria infection model [27] and Kaba *et al.*[11], in a year-long study demonstrated 100% protection of mice against sporozoite challenge if SAPN-induced *Pf*CSP-specific serum antibodies remained above 7.3 μg/ml.

The ultimate power of this humoral response, however, was demonstrated by the ability of *Pf*CSP-SAPN to induce antibodies that prevent invasion and inhibit the growth and development of *P. falciparum* sporozoites within human hepatocytes. Thus, this SAPN induced humoral response appears to be acting to: (1) suppress parasite motility; (2) induce the activation of the classical pathway of C’ for sporozoite lysis; and, (3) prevent the invasion and growth of parasites in cultured human liver hepatocytes.

The requirement for C’ is not absolute. Clearly the literature has demonstrated anti-CSP monoclonal antibodies, such as the positive control NSF-1 monoclonal used in the ILSDA reported here work without C’. But it must be emphasized that the selection of these monoclonal antibodies did not include C’ in the process.

An intriguing observation, however, is that even without this humoral response, SAPN-induced cellular immunity is able to prevent blood-stage malaria infection [11]. There are several factors that can aid in strong and long-lived immune responses to antigen. Variables such as mitogenicity, dosing and exposure times can all affect the robustness of resulting host immunity. The SAPN is about 35–40 nm in diameter, which allows it to easily pass through the lymphatic system without the need to be picked up and carried to the lymph nodes by antigen presenting cells (APC). Once in the lymph node, the exogenously-derived nanoparticles can be taken up by the local predominant APC, the dendritic cell (DC), and processed for loading onto MHC class I molecules of CD8+ T-lymphocytes via a process known as cross-presentation. The classical processing of SAPN could be accounted for by the presence of the universal pan CD4 helper epitope, PADRE, which is included in the *Pf*CSP-SAPN vaccine [11]. As little evidence has been shown to demonstrate absolute requirements for cross-presentation of proteins, only a few things are commonly agreed on as important in this process.

In the SAPN vaccine construct denoted as *Pf*CSP-KMY-SAPN, three CD8+ T-cell epitope peptides derived from the *Pf*CSP, in addition to the B-cell targeted epitopes of the *Pf*CSP repeat region, were used [11]. It is known that the CSP aids in liver stage invasion and development within the host, and it is believed that induction of a primed CD8+ T-cell response in the liver results in destruction of parasites that survived from the point of skin infection to liver infiltration. The mechanisms by which exogenously-delivered peptides are presented by the APC to the T-cell antigen receptor of CD8+ T-lymphocytes to induce T-cell priming has been one of the great enigmas of immunology. Cross-presentation goes against the dogma that primarily endogenously derived protein epitopes are loaded to MHC class I molecules. If efficient presentation of exogenously derived antigen could be induced, it would enable highly efficient and robust responses to multiple vaccination schemes and result in enhanced host immunity. Although the nuances of SAPN function are not yet completely understood, it is possible that the ordered array of peptide sequences, the hydrophobic/hydrophilic nature and/or physical structure intrinsic to the SAPN design, as well as the dose of antigen are all unique and effective at enabling cross-presentation of SAPN peptides to the immune system. Through the prolonged segregation and processing of SAPN, and the recruitment of TAP to the EE, there is a much stronger stimulation over an extended period of time than that observed with classical soluble proteins, such as ovalbumin. These observations give a much better understanding of what mechanisms are triggered by SAPN to induce and refine the long-lived adaptive cellular immune responses observed following SAPN vaccination [10,11].

In light of the confocal and TEM results presented here, a model for the cross-presentation and processing of SAPN within DC that may ultimately be responsible for the long-lived B- and T-cell responses observed following SAPN vaccination is put forward as a possible explanation. There are several proposed models that describe the complex mechanisms behind cross-presentation, we promote here that the observed delayed and segregated SAPN processing, as well as the recruitment of TAP to early endosome organelles is a mechanism of cross-presentation taken by SAPN. This enabled slow and continuous presentation of antigen to the adaptive immune system. Compared to the traditional model of direct shuttling of cytoplasmic peptide to the endoplasmic reticulum for loading onto MHC class I molecules, this model of peptide processing promotes induction of long-lived cellular responses to vaccine-delivered peptides. Further processing of SAPN does appear to occur at the lysosome/endoplasmic reticulum stage, which follows the accepted model that CD4+ T-cell-predicted peptides are processed and presented via MHC class II molecules in a classical manner. Thus, evidence is presented here in support of a mechanism for cross-presentation which results in the presence of long-lived memory CD8+ T-lymphocytes as demonstrated previously [11].

Previous work has examined and proven the SAPN platform as a viable method for inducing long-lived and effective adaptive host immune responses in mice via the delivery and processing of parasite antigens. Potential improvements to this vaccine could be made with the addition of an adjuvant in order to boost the magnitude of the responses if needed in higher order mammals. Also, the inclusion of sequences from other malaria strains, such as the *P. vivax* CSP repeat sequence; or epitopes from various *P. falciparum* proteins encompassing several of the parasite life stages into a single platform could enhance the scope of immunity.

## Abbreviations

SAPN: Self-assembling protein nanoparticle; CSP: Circumsporozoite protein; EDTA: Ethylenediaminetetraacetic acid; TAP: Transporter associated with antigen processing; EE: Early endosome; BMDDC: Bone marrow-derived dendritic cells; C’: Complement; ILSDA: Inhibition of liver stage development assay; CPHH: Cryopreserved primary human hepatocytes; TEM: Transmission electron microscopy; DLS: Dynamic light scattering; IFA: Immunofluorescence assay; APC: Antigen presenting cell; DC: Dendritic cell; MHC: Major histocompatibility complex; PBS: Phosphate buffered saline.

## Competing interests

PB has an interest in the company Alpha-O Peptides that has patents or patents pending on the SAPN technology.

## Authors’ contributions

MEM, SAK and DEL contributed to the design of the experiments; MEM, HEG, SAK, performed the experiments; TAPFD and PB designed the plasmids used to express the recombinant proteins that formed the nanoparticles; TAPFD and YY made the gold nanoparticles; MEM and DEL wrote the manuscript. All authors read, were involved in interpretation of results and approved the final manuscript.
