# Mechanisms of protective immune responses induced by the *Plasmodium falciparum* circumsporozoite protein-based, self-assembling protein nanoparticle vaccine

**DOI:** 10.1186/1475-2875-12-357

**Published:** 2013-10-07

**Authors:** Margaret E McCoy, Hannah E Golden, Tais APF Doll, Yongkun Yang, Stephen A Kaba, Xiaoyan Zou, Vincent R Gerbasi, Peter Burkhard, David E Lanar

**Affiliations:** 1 Malaria Vaccine Branch, WRAIR, 503 Robert Grant Avenue, Silver Spring, MD 20910, USA; 2 Institute of Materials Science, University of Connecticut, 97 North Eagleville Road, Storrs, CT 06269, USA; 3 Navy Medical Research Center, 503 Robert Grant Avenue, Silver Spring, MD 20910, USA; 4 Department of Molecular and Cell Biology, University of Connecticut, 97 North Eagleville Road, Storrs, CT 06269, USA

## Correction

After publication of this work [1], we noted that we inadvertently failed to include the complete list of all coauthors. The full list of authors has now been added and the Authors’ contributions and Acknowledgements section modified accordingly.

## Authors’ contributions

MEM, SAK, VRG and DEL contributed to the design of the experiments; MEM, HEG, SAK and XZ, performed the experiments; TAPFD and PB designed the plasmids used to express the recombinant proteins that formed the nanoparticles; TAPFD and YY made the gold nanoparticles; MEM and DEL wrote the manuscript. All authors read, were involved in interpretation of results and approved the final manuscript.
